# Host, vehicular and environmental factors responsible for road traffic crashes in a nigerian city: identifiable issues for road traffic injury control

**DOI:** 10.11604/pamj.2014.19.159.5017

**Published:** 2014-10-16

**Authors:** Peter Oladapo Adeoye, Dotun Musiliu Kadri, Jibril Oyekunle Bello, Chima Kingsley Pascal Ofoegbu, Lukman Olajide Abdur-Rahman, Adedeji Olugbenga Adekanye, Babatunde Akeeb Solagberu

**Affiliations:** 1 Centre for Injury Research and Safety Promotion (CIRASP), Department of Surgery, University of Ilorin Teaching Hospital, Ilorin, Kwara State, Nigeria; 2 Department of Surgery, Federal Medical Centre, Keffi, Nasarawa State, Nigeria; 3 Department of Surgery, Federal Medical Centre, Bida, Niger State, Nigeria; 4 Department of Surgery, Lagos State University Teaching Hospital, Ikeja Lagos State, Nigeria

**Keywords:** Road traffic injury, road traffic crash, crash mechanisms

## Abstract

**Introduction:**

Road traffic injury (RTI) has assumed major public health importance world-wide and the burden is heavier on the health-care infrastructure of countries in Sub-Saharan Africa. In Nigeria, RTI is the leading cause of trauma related morbidity and mortality. While there are some published epidemiological reports on RTI in the region, studies on the mechanism of causation of road traffic crashes (RTC) are not available.

**Methods:**

Over a 9-month period, we prospectively captured the 571 victims of RTC presenting to a single tertiary health care center in Nigeria. Data collected include demographic data, Mechanism of causation of RTC, Injuries sustained and outcomes.

**Results:**

Over three-quarters of the victims are young people and half were either traders (27.5%) or students (20%). Pedestrians, motorcycle riders and open truck occupants (people sitting at the rear loading compartment of trucks) often had fatal injuries. Analysis of collision patterns showed that lone crashes were the most frequent though car-to-motorcycle crashes caused a quarter of the deaths. Host factors (over-speeding driver, driver misjudgment, sleeping driver etc.) were responsible for four-fifths of the crashes while vehicular and environmental factors accounted for the remaining. On binary regression analysis, head injured victims had higher odds of dying than the non-head injured (Odds ratio = 6.5).

**Conclusion:**

This paper elucidates the mechanisms of causation of and types of injuries sustained following RTC in Nigeria and thus provide opportunities for prevention and control of this unacceptable situation.

## Introduction

Worldwide, Road Traffic Injury (RTI) has assumed major public health concern as it is responsible for an increasing number of morbidity and mortality. The World Health Organization (WHO) has attributed this increase to rapid urbanization and motorization and has aptly identified RTI as the price humanity must pay for modernization [1]. The cost burden is heavier in developing countries where modernization is approached with inappropriate road engineering and poorly implemented injury prevention programs. In Nigeria, published reports suggest that RTI is the leading cause of trauma related morbidity and mortality [2]. However, studies on mechanism of causation of RTI are few and far in between [1], thus; we evaluated the mechanisms of Road Traffic Crashes (RTC) amongst RTI victims who presented to our hospital with the aim of identifying potential causes and to make appropriate recommendations which should form the basis for improving or fine tuning injury prevention policies.

## Methods

**Figure 1:**
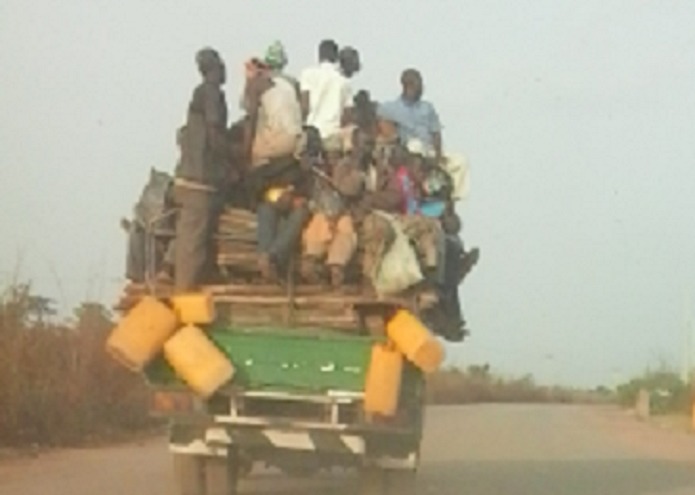
Open truck occupants. Scores of people sitting and standing at the rear compartment of a truck meant for loading goods. These are usually young traders who find this a cheap though dangerous mode of transportation to and from markets

All RTI victims who presented to University of Ilorin Teaching Hospital between October 2006 and June 2007 were prospectively captured. Biodata of victims, details of how the RTI occurred (mechanism of causation), the location of the victim during the incident (host status), the types of injuries sustained and eventual outcome was documented. Age categorization: The age of the victims were categorized into three groups (young 0-40 years; middle age 41-70 years and elderly >70 years) for statistical analysis. Host status: The host status was also categorized as riders of motorcycles (two wheeler vehicles); drivers of cars, buses or trucks (four wheeler vehicles); passengers of motorcycles; passengers of four wheeler vehicles; pedestrians and open truck occupants (Figure 1). Crash mechanism: Mechanisms leading to the crash were categorized into host, vehicle or environmental factors; this was adapted from Haddon's matrix [3]. Outcome: Injuries sustained and the immediate outcomes: alive and dead were also recorded. Analysis: Data analysis was performed using computer software SPSS version 16.0 package.

## Results

There were 571 patients over the nine month period consisting of 427 males and 144 females (M:F ratio = 3:1). There was no statistical significant difference in outcome between the sexes (p = 0.439). Median age was 30 years (range 6 months to 84 years). Over three-quarters of victims were young people, with another one-fifth being middle-aged and only 0.5% were elderly. There was however no significant statistical difference in outcomes between the age categories (p = 0.168). Majority of victims were either engaged in business or trading (27.5%) or were students (20%). The least involved group was the professional (2%). There were a total of 388 crashes consisting of 310 single patient and 78 mass (>1 victim) involvements (single: mass crash ratio = 4:1). Two crashes which involved 15 and 11 casualties constituted the highest mass crash casualty.

Pedestrians, motorcycle riders and open truck occupants (Figure 1) represent the worst hit hosts in% group mortality while passengers of 4 wheeled vehicles, motorcycle riders had the highest overall mortalities with both resulting in more than 60% of total recorded mortalities (Table 1). Four-wheeled vehicles were responsible for the highest number of casualties accounting for 286 (50%) patients; consisting of buses 170(59.4%), cars 88(30.8%) and lorries 28(9.8%). Motorcycles accounted for 162 (28.4%) casualties and accounted for the highest overall mortality of almost 39% against 35.4% form 4 wheeled vehicular crashes. Group mortality was highest amongst motorcycle (13.8%) and pedestrian (11.8%) crashes.

**Table 1:** The host status of RTI victims

Host type	Morbidity	Crash frequency	Mortality	% group mortality	% overall mortality
Motorcycle riders	102	99	9	8.8	29
Drivers of 4 wheeled vehicles	42	42	1	2.4	3.2
Passengers on motorcycles	60	56	3	5.0	9.7
Passengers on 4 wheeled vehicles	244	123	10	4.1	32.2
Pedestrian	86	83	5	11.8	16.2
Open truck occupant	37	16	3	8.1	9.7
TOTAL	571	421	31	5.4	100

Of the various collision patterns, lone crashes (Solitary crash involving only one vehicle with no collision with another vehicle or pedestrian) were the most frequent on our roads accounting for about 29% of 388 crashes and were responsible for the highest casualty of 199 patients (35%) (Table 2). Car-to-motorcycle crashes caused 22.5% of deaths recorded while 16.1% resulted from car-to-car crashes. Pedestrians were most frequently involved in motorcycles and cars crashes causing injuries in 41 (7.2%) and 39 (6.8%) of patients respectively and collectively resulted in 16.2% of mortality recorded. Host factors were responsible for 74% of injuries and more than 80% of crashes (Table 3). They also resulted in the highest group and overall mortality of 6.2% and 84% respectively. There is no statistical difference in outcome for the groups compared (p = 0.523). Data also showed that vehicle factors were due to burst tires 44 (42.7%), break failure 40 (39.8%), dislodged wheel 13 (12.6%), absence of reflectors 5 (4.9%), and jammed steering wheel 1 (1%). Host factors included over-speeding 336 (80%), driver misjudgment 40 (9.5%), sleeping driver 17 (4%), passenger misjudgment 12 (2.9%), pedestrian misjudgment 5 (1.2%), deliberate escape 3 (0.7%), intoxicated driver 3 (0.7%), intoxicated pedestrian 2 (0.5%), open truck occupant 2 (0.5%), and misadventure 1 (0.2%). Potholes accounted for majority of the environmental factors 24 (63.2%). Others are stationary vehicle/objects 8 (21.1%), misadventures 4 (10.5%), and absence of road shoulders 2 (5.3%).

**Table 2:** Collision pattern of crashes

Collision pattern	Morbidity	Frequency	Mortality	% group mortality	% overall mortality
Car-Lorry	28	13	2	7.1	6.5
Car-Car	30	18	5	16.7	16.1
Car-Mc	82	68	7	8.5	22.5
Car-Pedestrian	39	37	3	7.7	9.7
Lorry-Lorry	12	5	1	8.3	3.2
Lorry-Pedestrian	5	5	0	0	0
Lorry-Mc	13	10	2	15.3	6.5
Mc-Mc	27	23	3	11.1	9.7
Mc-Pedestrian	41	39	2	4.9	6.5
Lone crash	199	111	3	1.5	9.7
Bus-Bus	22	11	1	4.5	3.2
Bus-Lorry	18	5	1	5.5	3.2
Bus-Pedestrian	6	6	1	16.7	3.2
Bus-Car	17	8	0	0	0
Non-crash	25	23	1	4.0	3.2
Not known	7	6	0	0	0
TOTAL	571	388	31	5.4	100

**Table 3:** Causative factor of crashes

Problem	Morbidity	Frequency	Mortality	% group mortality	% overall mortality
Vehicle	103	46	3	2.9	9.7
Host	421	314	26	6.2	83.9
Environment	38	20	2	5.2	6.5
Not known	9	8	0	0	0
TOTAL	571	388	31	5.4	100

Soft tissue injuries constituted the largest (35%) injuries (Table 4). Head injury occurred solitarily or in combination in 168 patients (29.4%) resulting in 35.5% as single cause and 58% in multiple injuries. When injured victims of the RTI were re-classified as head injured and non- head injured and analyzed, there was statistical significant difference in outcome between the two groups (p= 0.000). On binary regression analysis head injured victims had a 6.5 times increased odds of dying than those non-head injured (p = 0.000, B= 1.884).

**Table 4:** Types of injury sustained by victims

Injury	Frequency	% Injury	Mortality	% group mortality	% overall mortality
Head injury	100	17.5	11	11.0	35.5
Limb fractures	130	22.8	3	2.3	9.7
Head + Limb fractures	59	10.3	7	11.9	22.6
Soft tissue injury	199	34.9	2	1.0	6.5
Blunt chest injury	26	4.6	0	0	0
Abdominal injury	7	1.2	0	0	0
Head + Chest/Abdominal injury + Limb fractures	5	0.9	0	0	0
Head + Chest/Abdominal injury	5	0.9	0	0	0
Chest/Abdominal injury + Limb fractures	7	1.2	0	0	0
Spine injury	4	0.7	0	0	0
Burns	7	1.2	0	0	0
Others	4	0.7	0	0	0
None	7	1.2	0	0	0
Unknown	11	1.9	8	72.7	25.8
TOTAL	571	100	31	5.4	100

## Discussion

The African region has been identified to carry the highest road traffic death rate of all continents [1, 4]. South Africa and Nigeria account for most of the reported deaths from Sub-Saharan Africa with values of 9,000 and 6,185 per year respectively [5]. These figures are likely to be higher considering the level of under-reporting in Africa when compared to developed countries. Two-thirds of victims being males and about 73% being active young adults (11-40 years) from this study underscores the economic impact this has on the society. Road crashes have been documented as a cause of poverty [4]. The loss or incapacitation of a bread winner is catastrophic as he is not only responsible to his nuclear family but also the extended family in a typical Nigerian setting. Previous reports documented similar vulnerable population group with about 50% fatality and 59% disability adjusted life years lost due to RTI [4, 6].

About 43% of victims in this study were passengers of 4 wheeled vehicles, 28.4% were on motorcycles (rider and passengers), and 14.5% were pedestrians. These three groups of road users are particularly prone to RTI in the developing countries [4–7]. The motorcycle has proved to be a dangerous mode of transportation with the highest mortality of almost 39%, yet there is increasing use of this mode of transportation because it is cheaper to acquire and easier to meander heavy traffic in urban regions [8]. In this study, none of the motorcyclist wore protective helmet. The risks posed by motorcycles have previously been reported from this centre [8]. The mini-bus is the major mode of inter-city transportation for the low and middle income class in Nigeria. These buses are mostly second-hand imports from wealthier countries and mostly lack up-to-date safety features [7]. They are mostly utilized by small and medium scale business entrepreneurs and by students. It is not surprising that these were the two occupational groups mostly affected. The professionals, most of who would travel in private vehicles were least involved (2%). Though these private vehicles are also mostly second-hand, they are better maintained. Despite the enactment of seat belt law in Nigeria, none of the victims used seat belts. Most commuter vehicles do not have seat belts in them and are loaded beyond passenger capacity which prevents the use of seat belts even when they are available. A new category of victims seen are open truck occupants; these are passengers travelling not in passenger compartment but on the back of trucks and lorries. These groups are often traders travelling with their goods and often sitting atop the goods at the back of the vehicles. During RTI, they are often thrown off the vehicles and their loads thrown right on top of them causing severe injuries. This group had the third highest group mortality rate of 8.1% in this study (Table 1).

Eighty percent of crashes were due to host factors and accounted for 74% of morbidity and about 84% of mortality. Over-speeding/inappropriate speed constituted the major host problem (80%). Odero's presentation also identified human error as leading cause with over-speeding topping the list in Kenya, Uganda, South Africa, Ghana, Zimbabwe, Ethiopia and Tanzania [5]. Alcohol intoxication constituted minor significance (1.2%) when compared to developed countries. Huston et al reported up to 62% of alcohol involvement when they evaluated police pursuit fatalities in USA [9]. In Nigeria, most commuters use fairly used (commonly referred to as “Tokunbo”) tires. This probably is the reason why burst tires are the leading cause of vehicular factor resulting in RTI unlike in Saudi Arabia where it was attributed to intense heat [10]. Forty percent being due to brake failure raises question as to adequacy of maintenance of the vehicles. Of the environmental factors, potholes (63.2%) and stationary vehicle/objects (21.1%) were the leading culprits. Design and engineering of our roads is sub-optimal. Potholes are not filled promptly and are left to get deeper. Broken down vehicles are not evacuated promptly and scenes of refuse tanks sited on major roads are common. Though soft tissue injury is the leading cause of morbidity, head injury significantly increased the odds of dying. A similar pattern has been previously reported from this center [2]. Use of helmets by cyclists and seatbelts by passengers of 4 wheeled vehicles and a law against travelling on the back of trucks or lorries may reduce the occurrence and /or severity of head injuries.

The following recommendations are suggested to policy makers to reduce the occurrence of and injuries from RTI: 1) Enforcement of helmet use by motorcyclists, seat belts by drivers and passengers of 4 wheeled vehicles and a law against travelling on the back of trucks and Lorries. 2) Strict regulation and enforcement of speed limit. The Federal Road Safety Corps should be equipped with speed detectors in their patrol cars and our roads must have speed detectors at strategic points with camera facilities. 3) Appropriate road engineering with road signs and conspicuously displayed speed limits to guide road users. Provision of road shoulders and service lanes will prevent broken down vehicles from being impediments to traffic. 4) Construction of new roads to open new network of roads so as to decongest the major roads. 5) Regular road maintenance to eliminate potholes. 6) House to house waste collection coordinated by the local government authority should be introduced to replace the current practice of refuse tanks dumped on major roads. 7) Use of “Tokunbo” tires must be banned urgently.

## Conclusion

This paper has elucidated the mechanisms involved in causation of RTIs, the injury spectrum seen in involved victims and the relationships of these variables to outcome of clinical management of the victims. Several opportunities for control are also put forward based on the findings in the study.
